# A Neural Network Model Using Pain Score Patterns to Predict the Need for Outpatient Opioid Refills Following Ambulatory Surgery: Algorithm Development and Validation

**DOI:** 10.2196/40455

**Published:** 2023-02-08

**Authors:** Rodney Allanigue Gabriel, Sierra Simpson, William Zhong, Brittany Nicole Burton, Soraya Mehdipour, Engy Tadros Said

**Affiliations:** 1 Department of Anesthesiology University of California San Diego La Jolla, CA United States; 2 Department of Anesthesiology and Perioperative Medicine University of California Los Angeles Los Angeles, CA United States

**Keywords:** opioids, ambulatory surgery, machine learning, surgery, outpatient, pain medication, pain, pain management, patient needs, predict, algorithms, clinical decision support, pain care

## Abstract

**Background:**

Expansion of clinical guidance tools is crucial to identify patients at risk of requiring an opioid refill after outpatient surgery.

**Objective:**

The objective of this study was to develop machine learning algorithms incorporating pain and opioid features to predict the need for outpatient opioid refills following ambulatory surgery.

**Methods:**

Neural networks, regression, random forest, and a support vector machine were used to evaluate the data set. For each model, oversampling and undersampling techniques were implemented to balance the data set. Hyperparameter tuning based on k-fold cross-validation was performed, and feature importance was ranked based on a Shapley Additive Explanations (SHAP) explainer model. To assess performance, we calculated the average area under the receiver operating characteristics curve (AUC), F1-score, sensitivity, and specificity for each model.

**Results:**

There were 1333 patients, of whom 144 (10.8%) refilled their opioid prescription within 2 weeks after outpatient surgery. The average AUC calculated from k-fold cross-validation was 0.71 for the neural network model. When the model was validated on the test set, the AUC was 0.75. The features with the highest impact on model output were performance of a regional nerve block, postanesthesia care unit maximum pain score, postanesthesia care unit median pain score, active smoking history, and total perioperative opioid consumption.

**Conclusions:**

Applying machine learning algorithms allows providers to better predict outcomes that require specialized health care resources such as transitional pain clinics. This model can aid as a clinical decision support for early identification of at-risk patients who may benefit from transitional pain clinic care perioperatively in ambulatory surgery.

## Introduction

Opioids play an essential role in acute perioperative pain management. Increased attention to pain management as a quality metric has brought to light an overuse of prescription opioids contributing to an epidemic across the United States. The United States has had increased opioid prescriptions filled in the immediate postoperative period; a study reported that the mean dose of opioids prescribed for most surgical procedures in the United States was higher than that prescribed in other countries [1]. Opioid-related deaths made up one of the largest causes of preventable deaths in the United States, which costed an estimated US $78.5 billion to US $1.02 trillion to the US health care system [2,3].

Persistent opioid prescribing is often postsurgical [4], in which as many as 3% of opioid-naive patients required opioids for more than 90 days after a major elective surgery [5]. One potential service that may help curb outpatient opioid use after surgery is the transitional pain clinic, which consists of a team of providers who implement multidisciplinary opioid-sparing approaches such as pharmacological, nonpharmacological, and psychological interventions with the goal of weaning patients from opioids postoperatively as outpatients [6,7]. Transitional pain clinics have been shown to reduce opioid use postoperatively, symptoms of anxiety and depression, pain catastrophizing, and postsurgical pain [8,9]. Given the increased resources required to provide this type of service, not all surgical patients may realistically receive postoperative care from transitional clinics. Currently, the criteria for recommendation to transitional pain services for surgical patients are not uniformly defined; thus, accurate predictive methods for patients who may benefit from transitional pain clinics are needed. Less work has been done on patients undergoing ambulatory surgery and on the identification of patients who may likely require more opioids as an outpatient. In such populations, machine learning may be used to identify postoperative opioid use in the recovery room [10]. In addition, some studies have described the risk factors for using outpatient opioids after ambulatory surgery [11-13].

The objective of this study was to develop machine learning–based predictive models that may aid in the identification of patients likely to require opioid refills after their initial discharge prescription. Specifically, pain score patterns were incorporated (ie, trends in reported pain scores in the recovery room) into the models. We focused on patients who underwent ambulatory surgery, which included orthopedic surgery (eg, joint arthroscopy, forearm/hand surgery), nonmastectomy breast surgery, urology (eg, cystoscopy), minimally invasive surgery (eg, cholecystectomy, hernia repair), colorectal surgery (eg, hemorrhoidectomy), and gynecology (dilation and curettage/evacuation, hysteroscopy). We hypothesized that the use of neural networks that incorporate various features, including recovery room pain phenotypes, may identify patients at higher risk. The pain phenotypes included patterns in patient-reported pain scores in the recovery room, including trajectory of pain and median and maximum pain scores.

## Methods

### Study Population

Data were retrospectively collected from the electronic medical records of patients who underwent outpatient surgery from January to July 2020 at a single ambulatory surgery center. The outpatient surgeries included in the analysis included orthopedic surgery (eg, joint arthroscopy, forearm/hand surgery), nonmastectomy breast surgery, urology (eg, cystoscopy), minimally invasive surgery (eg, cholecystectomy, hernia repair), colorectal surgery (eg, hemorrhoidectomy), and gynecology (dilation and curettage/evacuation, hysteroscopy).

### Ethics Approval

Our institutional review board (Human Research Protections Program) waived the consent requirement and approved this retrospective study (protocol 210099).

### Primary Outcome and Features

The primary outcome of interest was a binary variable (response range, 0 or 1), in which 0 was defined as “no opioid refill” and 1 was defined as the patient “needed to refill their outpatient opioid prescriptions” within 2 weeks after surgery (no opioid refill vs opioid refill). This was captured retrospectively from the electronic medical record by review of the following: (1) any telephone note describing patient calling in for opioid renewal; (2) any progress/office visit note from primary care provider, pain medicine specialist, or surgical provider describing the need for opioid refill; or (3) any renewal order in the medication list for opioids within this time frame. On postanesthesia care unit (PACU) discharge, all patients were prescribed up to 5 days of opioids. For perioperative multimodal analgesia, all patients received preoperative acetaminophen unless contraindicated. For a subset of surgical procedures, regional nerve blocks were routinely offered preoperatively (ie, shoulder, hand/forearm, and knee surgeries). Intraoperatively, patients may have received fentanyl, hydromorphone, ketamine, ketorolac, and dexmedetomidine at the discretion of the anesthesiologist. In the PACU, patients were given oxycodone, fentanyl, and hydromorphone, as needed.

Features that were integrated into the model were collected retrospectively from the electronic medical record system. The data included age (years), sex (male vs female), body mass index (kg/m^2^), English-speaking, comorbidities, regional nerve block performance, general anesthesia, intraoperative ketamine, intraoperative total intravenous anesthesia, opioid consumption, and pain scores (11-point numeric rating scale [NRS] from 0 to 10). These features were included, as they were determined to be relevant to postoperative opioid use based on clinical judgement and previous research [14,15]. Opioid consumption, defined as total opioids consumed intraoperatively and in the PACU, was measured in intravenous morphine equivalents (MEQ). Pain scores were captured as preoperative pain score, median pain score in the PACU, maximum pain score in the PACU, and slope of pain score trajectory in the PACU. Preoperative pain scores were collected by nurses upon arrival for preoperative check-in. PACU pain scores were captured every 5-15 minutes and recorded in the electronic medical record. A negative value for the pain score slope was defined as an overall decrease in pain scores throughout the PACU stay. A positive value for the pain score slope was defined as an overall increase in the pain scores throughout the PACU stay. A zero value of the pain score slope was defined as no change in the overall trend of pain scores throughout the PACU stay.

### Statistical Analysis

Python (v3.10.1) was used for all statistical analyses. Patient and surgical characteristics were compared with chi-squared test (categorical) and Wilcoxon rank sum test (continuous). A generalized linear mixed model fit by maximum likelihood was implemented to model the features to the primary outcome of opioid refill. The random effect in this model was the surgical procedure. All features were included in the model, and their association with the outcome was reported by their respective odds ratios (ORs), 95% CI, and *P* values. A neural network model to predict the need for opioid refills following surgery was constructed. Logistic regression, random forest, and support vector machine classifiers were implemented for comparison. For all models, patient data were divided into training and test data sets with a 70:30 split by using a stratified randomized splitter—the train_test_split method from the sci-kit learn library. K-fold cross-validation on the training set was used to tune the hyperparameters and to optimize oversampling techniques as well as to calculate the average sensitivity, specificity, F1-score, and area under the curve (AUC) for the receiver operating characteristic curve. The final version of each model was then validated on the test set and the AUC was reported. Feature importance from the neural network was ranked based on Shapley Additive Explanations (SHAP).

### Data Balancing

Synthetic Minority Oversampling Technique (SMOTE) for Nominal and Continuous algorithm and random undersampling were both implemented using the imblearn library [16]. These tools were used to achieve a balanced class distribution with minimal difference between positive and negative outcomes. A data set with a large difference between positive and negative outcomes was considered unbalanced and may make it difficult for predictive machine learning models to draw useful conclusions, given the uneven classification of data.

Random undersampling of the majority outcome is frequently used to reduce the impact of imbalanced data sets; however, SMOTE oversamples were used to create synthetic minority class examples to balance the minority class with the majority class. SMOTE uses samples from the minority class and a set number of nearest neighbors—in this case, 5—to generate synthetic cases from the sample class. Combining the 2 techniques as outlined yielded positive outcomes. Both techniques were only applied on the training set. Different combinations for proportions of minority to majority class were analyzed, ranging from 0.25 to 1.00. After performing k-fold cross-validation, the parameter “sampling_strategy” for the SMOTE class from imblearn was optimal when set to 0.24 and the parameter “sampling_strategy” for the RandomUnderSampler class from imblearn was optimal when set to 0.94. Optimal results were based on which hyperparameters produced the highest performance metrics for the model (eg, AUC, F1-score, sensitivity, specificity).

### Machine Learning Models

Four different machine learning classification models were evaluated: neural network, logistic regression, random forest classifier, and support vector classifier. For each model, the following sampling methods were compared: oversampling the training set via SMOTE, undersampling the majority class in the training set, a combination of SMOTE and undersampling of the majority classes, and no oversampling or undersampling technique. The results from the sampling method that provided the optimal results were reported. For each model, all features were included as inputs. One-hot encoding was used for categorical features.

#### Multilayer Perceptron Neural Network

Using the Keras interface for the TensorFlow library, a shallow feedforward neural network was constructed. The rectified linear unit function was used as the activation function. The final output layer used the sigmoid activation function, and the overall model used the Adam optimizer. Repeated k-fold cross-validation was used to tune the hyperparameters to find the optimal parameter values for the number of hidden layers (1), number of neurons per hidden layer (100), maximum number of iterations (300), batch size (16), and learning rate (0.0001).* *

#### Logistic Regression

The logistic regression classifier predicts the probabilities of the different outcome possibilities based on the input. A newton-cg solver regression model was implemented without specifying individual class weights. This model provided a baseline score and helped make the case for improvement over the evaluation metrics. Repeated k-fold cross-validation was used to tune the hyperparameters to find the optimal parameter value for *C* (the strength of the regularization is inversely proportional to *C*), which was 0.3.

#### Random Forest Classifier

The random forest is an ensemble approach, which has been proven effective for a variety of classification problems. To tune the hyperparameters, we performed repeated k-fold cross-validation to find the optimal parameter values for maximum depth (75), minimal samples required to be at the leaf node (4), minimal samples required to split an internal node (4), and number of estimators (100) (ie, number of trees).

#### Support Vector Classifier

A support vector classifier maps the data onto an n-dimensional space (n being the number of features) and then identifies the hyperplane decision boundary that best separates the data into 2 classes by maximizing the distance between the hyperplane and the nearest data point in either class. K-fold cross-validation was used to tune the hyperparameters to identify the optimal parameter value for *C*, which was 130. 

### Performance Metrics

The primary performance metric of interest was the AUC for the receiver operating characteristic curve. In addition, we reported F1-scores, sensitivity, and specificity.

### K-Fold Cross-Validation

To effectively tune the hyperparameters of the models, stratified k-fold cross-validation was implemented on the training set to observe the sensitivity, specificity, F1-score, and AUC scores, for 10 splits. For each iteration, the data set was split into 10 groups (folds). One fold served as the test set with the other 9 serving as the training set. When assessing the effectiveness of SMOTE and random undersampling, only the training folds were changed. This process was repeated until each fold served as the test set once. Every model exhibited improved performance metrics when SMOTE and random undersampling were applied.

## Results

### Study Cohort Characteristics

There were 1333 patients and 28 unique surgical procedures included in the final analysis, and 144 (10.8%) patients refilled their opioid prescription within 2 weeks after outpatient surgery. Univariate analysis revealed that patients who required opioid refills were more likely to be smokers (32/144, 22.2% vs 156/1189, 13.1%, respectively; *P*=.005) and had a regional nerve block performed (87/144, 60.4% vs 440/1189, 37%, respectively; *P*<.001). Those who required opioid refills had higher total perioperative opioid consumption (intraoperative and PACU opioid use; *P*<.001), preoperative pain scores (*P*<.001), maximum PACU pain scores (*P*<.001), and median PACU pain scores (*P*<.001). Table 1 lists the differences between the opioid refill and non–opioid refill cohorts represented in the study population in order to provide information regarding the baseline characteristics. All surgical procedures included in our analysis are listed in Table 1.

**Table 1:** Baseline characteristics of the study cohorts.^a^

Feature		No opioid refill (n=1189)	Required opioid refill (n=144)	*P* value	
**Surgical procedure, n (%)**					<.001
	Arthrodesis (finger)	10 (0.8)	3 (2.1)		
	Arthroscopy (hip)	36 (3)	5 (3.5)		
	Arthroscopy (knee)	99 (8.3)	12 (8.3)		
	Arthroscopy (shoulder)	61 (5.1)	19 (13.2)		
	Arthroscopy (shoulder, with rotator cuff repair)	45 (3.8)	13 (9)		
	Arthroscopy (wrist)	9 (0.8)	4 (2.8)		
	Breast lumpectomy	56 (4.7)	7 (4.9)		
	Transperineal prostate biopsy	17 (1.4)	0 (0)		
	Laparoscopic cholecystectomy	29 (2.4)	1 (0.7)		
	Cystoscopy	43 (3.6)	0 (0)		
	Dilation and curettage of the uterus	76 (6.4)	0 (0)		
	Dilation and evacuation of the uterus	36 (3)	0 (0)		
	Anorectal examination under anesthesia	29 (2.4)	0 (0)		
	Condyloma excision	13 (1.1)	3 (2.1)		
	Lesion excision of the head and neck	31 (2.6)	1 (0.7)		
	Lesion excision of the upper extremity	78 (6.6)	4 (2.8)		
	Extracorporeal shockwave lithotripsy	20 (1.7)	1 (0.7)		
	Anal fistulectomy	73 (6.1)	1 (0.7)		
	Hemorrhoidectomy	29 (2.4)	8 (5.6)		
	Inguinal herniorrhaphy	16 (1.3)	2 (1.4)		
	Hysteroscopy	47 (4)	0 (0)		
	Incision and drainage of the upper extremity	12 (1)	0 (0)		
	ORIF^b^, distal radius fracture	71 (6)	15 (10.4)		
	ORIF, scaphoid fracture	11 (0.9)	0 (0)		
	ORIF, hand	70 (5.9)	10 (6.9)		
	Ligament reconstruction with tendon interposition, upper extremity	30 (2.5)	9 (6.3)		
	Open carpal tunnel release	50 (4.2)	2 (1.4)		
	Arthroscopic anterior crucial ligament repair	92 (7.7)	24 (16.7)		
**Patient characteristics **					
	Age (years), median (quartiles)	43 (32, 60)	46.5 (33.0, 58.25)	.67	
	Male sex, n (%)	551 (46.3)	70 (48.6)	.59	
	Body mass index (kg/m^2^), median (quartiles)	26.1 (23.0, 30.2)	26.3 (23.9, 31.6)	.14	
	English speaker, n (%)	1106 (93)	129 (89.6)	.19	
**Comorbidities, n (%) **					
	Preoperative opioid use	55 (4.6)	8 (5.6)	.77	
	Diabetes mellitus	87 (7.3)	13 (9)	.57	
	Alcohol use	53 (4.5)	7 (4.9)	.99	
	Active smoker	156 (13.1)	32 (22.2)	<.001	
	Substance abuse	43 (3.6)	10 (6.9)	.09	
	Hypertension	255 (21.4)	34 (23.6)	.63	
	Depression/anxiety	246 (20.7)	37 (25.7)	.21	
	Coronary artery disease	35 (2.9)	3 (2.1)	.78	
	Dementia	10 (0.8)	1 (0.7)	.99	
	Renal insufficiency	40 (3.4)	4 (2.8)	.91	
	Chronic obstructive pulmonary disease	18 (1.5)	2 (1.4)	.99	
	Asthma	116 (9.8)	16 (11.1)	.71	
	Obstructive sleep apnea	84 (7.1)	9 (6.3)	.95	
	Chronic pain	114 (9.6)	18 (12.5)	.34	
	Congestive heart failure	9 (0.8)	0 (0)	.61	
**Anesthesia/perioperative medications **					
	Regional block performed, n (%)	440 (37)	87 (60.4)	<.001	
	General anesthesia, n (%)	783 (65.9)	107 (74.3)	.05	
	Intraoperative ketamine used, n (%)	55 (4.6)	9 (6.3)	.51	
	Total intravenous anesthetic, n (%)	43 (3.6)	6 (4.2)	.92	
	Total (intraoperative and PACU^c^) opioid consumption (MEQ^d^), median (quartiles)	10 (0, 17)	12.8 (6.9, 26)	<.001	
**Pain score (numeric rating scale 0-10), median (quartiles) **					
	Preoperative pain score	0 (0, 3)	0 (0, 6)	<.001	
	Maximum PACU score	2 (0, 6)	6 (0, 8)	<.001	
	Median PACU pain score	0 (0, 4)	3 (0, 6)	<.001	
	Slope of PACU pain	0 (–0.14, 0)	0 (–0.46, 0)	<.001	

^a^*P* values were calculated by chi-squared and Wilcoxon rank sum tests for categorical and continuous variables, respectively.

^b^ORIF: open reduction and internal fixation.

^c^PACU: postanesthesia care unit.

^d^MEQ: morphine equivalents.

### Mixed Effects Logistic Regression Model

The need for opioid refill within 2 weeks after ambulatory surgery was modeled utilizing a mixed effects logistic regression analysis fit by maximum likelihood, in which the random effect was the surgical procedure (Table 2). Features that were statistically significantly associated with higher odds of need for opioid refill were active smokers (OR 1.99, 95% CI 1.19-3.31; *P*=.009), substance abuse history (OR 2.34, 95% CI 1.02-5.37; *P*=.04), regional block performed (OR 2.81, 95% CI 1.62-4.88; *P*<.001), total opioid consumption (MEQ mg) intraoperatively and in PACU (OR 1.03, 95% CI 1.01-1.06; *P*=.008), and median PACU pain score (NRS) (OR 1.19, 95% CI 1.04-1.36; *P*=.01). A feature that was significantly associated with decreased odds of opioid refill was English-speaking patients (OR 0.47, 95% CI 0.24-0.93; *P*=.03).

**Table 2:** Mixed effects logistic regression modeling of refills.^a^

Feature			Odds ratio (95% CI)		*P* value
Age (years)			0.99 (0.98-1.01)		.85
Male sex			0.96 (0.63-1.46)		.84
Body mass index (kg/m^2^)			1.01 (0.98-1.01)		.48
English speaker			0.47 (0.24-0.93)		.03
**Comorbidities**					
	Preoperative opioid use	0.70 (0.25-1.98)		.51	
	Diabetes mellitus	1.44 (0.67-3.09)		.36	
	Alcohol use	0.82 (0.33-2.03)		.66	
	Active smoker	1.99 (1.19-3.31)		.009	
	Substance abuse	2.34 (1.02-5.37)		.04	
	Hypertension	1.26 (0.73-2.18)		.41	
	Depression/anxiety	1.07 (0.66-1.73)		.79	
	Coronary artery disease	1.23 (0.30-5.00)		.78	
	Dementia	1.33 (0.09-18.80)		.84	
	Renal insufficiency	0.79 (0.24-2.58)		.99	
	Chronic obstructive pulmonary disease	0.99 (0.19-5.17)		.99	
	Asthma	0.99 (0.53-1.86)		.99	
	Obstructive sleep apnea	0.62 (0.26-1.44)		.27	
	Chronic pain	1.31 (0.62-2.79)		.48	
	Congestive heart failure	0		.99	
**Anesthesia/perioperative medications**					
	Regional block performed	2.81 (1.62-4.88)		<.001	
	General anesthesia	1.05 (0.56-1.94)		.88	
	Intraoperative ketamine used	1.17 (0.51-2.65)		.71	
	Total intravenous anesthetic	1.21 (0.46-3.18)		.71	
	Total (intraoperative and PACU^b^) opioid consumption (MEQ^c^)	1.03 (1.01-1.06)		.008	
**Pain (numeric rating scale 0-10)**					
	Preoperative pain score	0.95 (0.86-1.05)		.35	
	Maximum PACU score	1.02 (0.92-1.13)		.67	
	Median PACU pain score	1.19 (1.04-1.36)		.01	
	Slope of PACU pain	0.78 (0.56-1.09)		.16	

^a^Results of mixed effects logistic regression modeling need for opioid refill after ambulatory surgery. The random effect in this model was the surgical procedure.

^b^PACU: postanesthesia care unit.

^c^MEQ: morphine equivalents.

### Neural Network Approach to Predicting Opioid Refills

**Figure 1:**
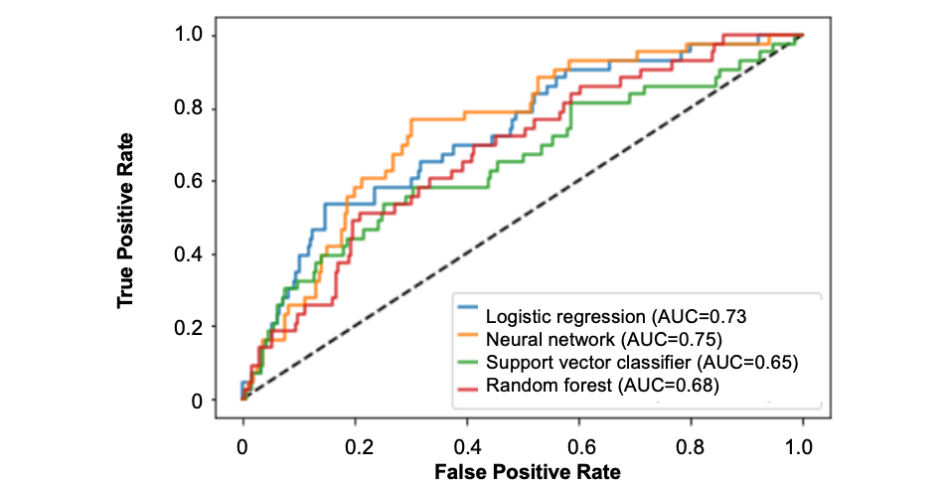
The calculated area under the curve of each machine learning model when trained on 70% of the data and validated on the remaining 30%. The models predict the need for opioid refill within 2 weeks following ambulatory surgery. AUC: area under the curve.

**Figure 2:**
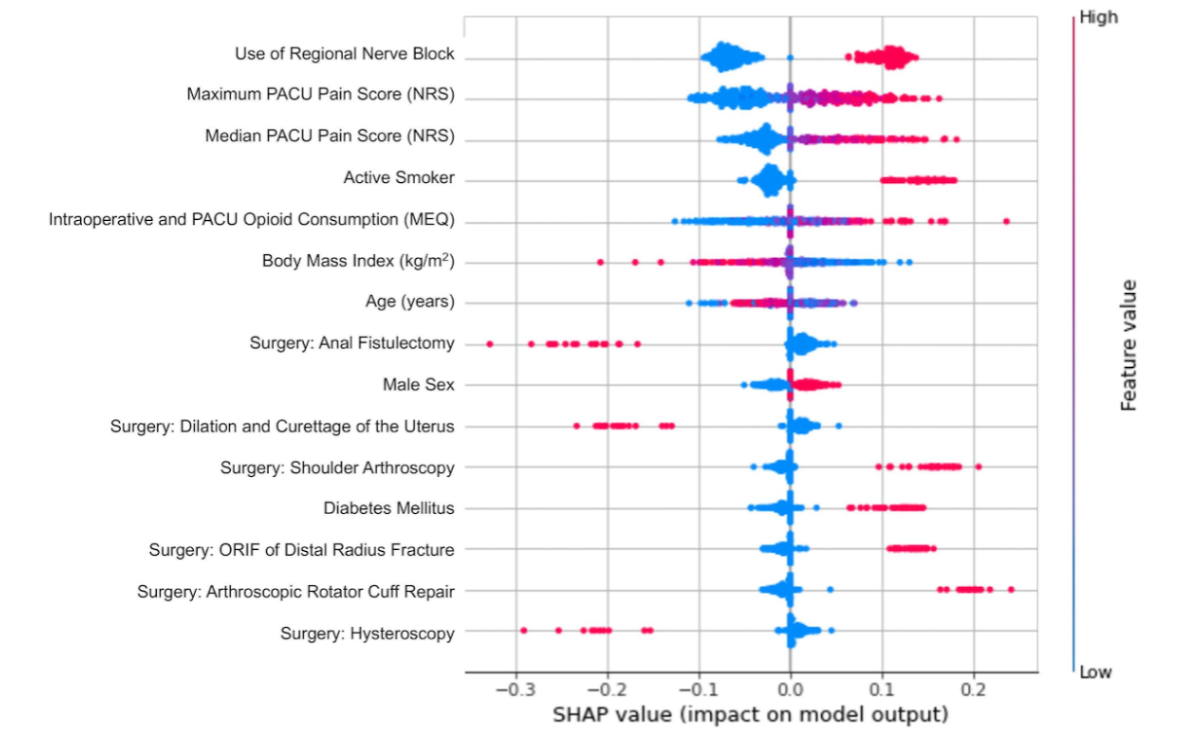
Shapley Additive Explanations (SHAP) values (impact on model output) of the top features used in the neural network predicting the need for opioid refill within 2 weeks following ambulatory surgery. MEQ: morphine equivalents; NRS: numeric rating scale; ORIF: open reduction and internal fixation; PACU: postanesthesia care unit.

Hyperparameter tuning via grid search cross-validation was implemented to identify the best architecture of the multilayer perceptron neural network, which consisted of 1 hidden layer, 100 neurons within the hidden layer, 300 maximum iterations for learning, a batch size of 16, and a learning rate of 0.0001. Based on this architecture, the average AUC calculated from k-fold cross-validation was 0.71 (95% CI 0.68-0.74). The final model was then validated on the test set, which yielded an AUC of 0.75 (Figure 1). The features with the highest impact on model output for the neural network based on the absolute SHA*P* values were performance of a regional nerve block, maximum pain score in the PACU, median pain score in the PACU, active smoking history, and total opioid consumption (intraoperative and PACU) (Figure 2).

Next, various other machine learning–based models were implemented to predict the need for opioid refills after ambulatory surgery. Based on k-fold cross-validation, the average AUCs from models with optimized hyperparameters were identified for support vector machine (0.64, 95% CI 0.57-0.71), random forest (0.66, 95% CI 0.60-0.71), and logistic regression (0.69, 95% CI 0.66-0.74) (Table 3). The final models for each machine learning approach were then validated on a separate test set when SMOTE was not applied versus when SMOTE was applied—the calculated AUCs were identified for support vector machine (0.65), random forest (0.68), and logistic regression (0.73). SMOTE improved the performance of each model.

**Table 3:** Model performance for each machine learning model calculated by k-fold cross-validation.

Classification model	Area under the curve			F1-score			Sensitivity			Specificity	
	No SMOTE^a^	SMOTE	No SMOTE		SMOTE	No SMOTE		SMOTE	No SMOTE		SMOTE
Logistic regression	0.516	0.698	0.033		0.335	0.040		0.673	0.992		0.722
Neural network	0.509	0.711^b^	0.067		0.347^b^	0.039		0.693^b^	0.979		0.726
Support vector classifier	0.500	0.643	0.001		0.291	0.002		0.602	0.999^b^		0.684
Random forest	0.500	0.655	0.002		0.317	0.001		0.524	0.999^b^		0.786

^a^SMOTE: Synthetic Minority Oversampling Technique.

^b^Represents the best performance for the given metric.

## Discussion

### Principal Findings

We demonstrated that a shallow feedforward neural network and other machine learning approaches that integrated pain score patterns had adequate performance to predict the need for opioid refills within 2 weeks following ambulatory surgery. The features with the highest impact on model output were active smoking history, intraoperative opioid consumption, PACU opioid consumption, regional nerve block utilization, as well as maximum and median pain scores in the PACU. The importance of pain score patterns (ie, median and maximum pain scores) in predicting opioid refills is interesting and highlights the association of PACU analgesia and opioid consumption with the requirement for more opioids following the initial prescription. This neural network may be useful in identifying patients at risk who require a longer duration of opioid use so that the limited hospital resources can be better utilized in a precise manner.

### Comparison to Prior Work

Previous studies have reported the utilization of machine learning for predicting postoperative opioid use in ambulatory surgery [10,17]. Nair et al [10] reported the accuracies of regression, naïve Bayes, neural networks, random forest, and extreme gradient boosting in predicting postoperative opioid use in the recovery room and showed that random forest performed best when using only preoperative features. Anderson et al [17] utilized models to predict prolonged opioid use specifically in patients who underwent anterior cruciate ligament repair by using regression, Bayesian belief network, gradient boosting, and random forest. They found that gradient boosting was able to achieve an AUC of 0.77. In our study, we reviewed multiple types of ambulatory surgeries and focused on predicting the need for additional outpatient opioid refills weeks after surgery. Four computational approaches were used to determine the best model for our data set, and all had similar performances. Random forest, logistic regression, and support vector machine tools did not perform as well as the neural network, though the random forest model had increased specificity compared to the neural network. Both the support vector classifier and neural network can increase the dimensionality of the data to find a solution, but given the time and training, neural networks usually outperform support vector classifiers. Random forest and neural networks approach data inversely, as random forest decision trees are independent and neural network neurons are dependent on other neurons. Logistic regression is the standard approach but often does not perform well in multidimensional data sets. By surveying multiple models, the benefits of each can be identified and evaluated to improve the validity of the predicted features [18].

Opioids remain the cornerstone for acute postoperative pain management, and the perioperative period is often the patients’ first introduction to prescription opioids. Our study’s patient cohort was primarily opioid-naïve; only 4.6% (55/1189) of the patients in the non–refill group and 5.6% (8/144) of the patients in the refill group reported preoperative opioid use. Studies have shown surgical procedure as an independent risk factor for prolonged opioid use [4,19,20]. Other risk factors include preoperative opioids, tobacco use, gender, and mood disorders [21-26]. Although efforts are in place to standardize postoperative opioid prescriptions per surgical procedure [27], there continues to be a wide variety in the amount and duration of opioids prescribed and often in excess [1,28-31].

An estimated 67%-92% of the prescribed opioids for postoperative pain remain unused [1,32], leaving great potential for diversion and misuse. An increasing number of heroin users reported first being introduced to opioids via prescription and then resorting to heroin for cost and availability factors [33]. Likewise, Bartels et al [34] report that 80% of the opioid prescriptions remain unused with limited and challenging disposal options. Similarly, a 2017 systematic review reports that patients took only 29%-58% of the prescribed opioid pills [32]. Over the decades, we have learned that excess opioids do not necessarily reduce persistent postsurgical pain or any other pain-related outcomes [35]. As Porter and colleagues [36] demonstrated, sometimes less is more—patient-centered opioid discharge prescription guidelines satisfied 93% of the patients, with 99% in the 0 morphine milligram equivalents group [36]. Although there may be some procedures that do not require postoperative opioids, we must also find a balance and prescribe opioids as necessary to meet individual patients’ pain needs [37]. For these reasons, risk stratification can be a helpful tool for guiding the process of postoperative opioid prescribing.

The use of regional anesthesia was associated with opioid refilling. It is important to note that there is no causality that may be drawn from these results but rather an association. It may be that the use of regional anesthesia was associated with surgeries that were more painful in nature, and despite pain scores being likely lower in the PACU, this group would more likely require additional opioids as outpatients when compared to other surgical procedures that are less likely to receive regional anesthesia for pain management. Other potential limitations include the variability in surgery type, which may range in pain level, both during surgery and during recovery, as well as the subjective nature of pain scores. Despite these limitations, the features that have been identified are actionable and trackable in future studies.

### Limitations

There are several limitations in this study—mainly due to the inherent limitations of a retrospective analysis. First, the primary outcome (opioid refills) may potentially be underestimated, as we captured this data based on clinical notes and orders in the electronic medical record system. It is possible that the need for opioid refills was missed in some patients who sought care outside of our health care system (and thus not recorded in our records). However, we extracted the data via a manual clinician review to optimize accuracy as much as possible. A prospective study would be needed to assess the incidence of postsurgical opioid refills more accurately. Second, an issue of generalizability is also of concern, as this is single-institution data. Model performance (eg, AUC) could decrease in a surgical population outside of this institution. To avoid the issue of overfitting and, thus, limited generalizability, we calculated the metrics from k-fold cross-validation and furthermore used a holdout data set for validation. What is needed is a high-quality prospective study that can more accurately capture the features and outcomes from each patient and, subsequently, be validated at external institutions.

### Future Directions

Early identification of at-risk patients prior to their elective surgical procedure is the key. These patients can then be referred to establish care with a dedicated and comprehensive transitional pain program. Built on solid evidence-based medicine, this multidisciplinary transitional pain service includes anesthesiology, pharmacy, psychiatry, and physical therapy. Patients are often evaluated preoperatively to help manage expectations regarding anticipated postoperative pain and offer preoperative weaning when appropriate. This anesthesiologist-led team makes recommendations about intraoperative and immediate postoperative pain management [38], including predischarge and postdischarge tapering plans, if applicable. After the discharge, the transitional pain service can continue to manage these patients by using a multimodal approach with nonopioid medication, interventional procedures such as regional peripheral nerve blocks [39] or cryoanalgesia [40], as well as provide necessary psychological support [41]. Transitional pain clinics have been shown to reduce opioid use postoperatively, symptoms of anxiety and depression, pain catastrophizing, and pain [7,8]. Early identification of these clinical predictors, in conjunction with knowing the typical pain trajectories and patterns of common surgical procedures [42], can serve as the foundation for the basis of prescribing the right regimen and duration for the opioid prescription. Anesthesiology as a specialty, and especially in the setting of a dedicated acute pain service, is well positioned to take the lead in defining personalized pain medicine through all 3 phases of perioperative care [43].

### Conclusions

Applying machine learning algorithms to electronic health data allows providers to develop models to predict more accurately and therefore appropriately allocate the limited health care resources (ie, transitional pain clinics). In this study, we showed that the need for regional anesthesia, high intraoperative opioid consumption, increased PACU pain scores, and opioid consumption were important features in models predicting outpatient opioid refills. Although providers are aware of the potential risk factors of opioid misuse, it remains challenging to accurately predict patients that will benefit from services as an outpatient. This prediction model serves as an example of a model that could be formalized into clinical decision support tools to help us better understand which patients will benefit from transitional pain clinics following ambulatory surgery.

## Abbreviations

AUC: area under the curve
MEQ: morphine equivalents
OR: odds ratio
PACU: postanesthesia care unit
SHAP: Shapley Additive Explanations
SMOTE: Synthetic Minority Oversampling Technique
